# Regeneration and Agrobacterium-mediated genetic transformation of twelve *Eucalyptus* species

**DOI:** 10.48130/FR-2022-0015

**Published:** 2022-11-24

**Authors:** Tingting Zhou, Ying Lin, Yan Lin, Jianzhong Luo, Jihua Ding

**Affiliations:** 1 College of Horticulture and Forestry, Hubei Hongshan Laboratory, Hubei Engineering Technology Research Center for Forestry Information, Huazhong Agricultural University, Wuhan 430070, China; 2 Research Institute of Fast-growing Trees (RIFT)/China Eucalypt Research Centre (CERC), Chinese Academy of Forestry (CAF), ZhanJiang, GuangDong, China

**Keywords:** *Eucalyptus*, Genotype, Regeneration, Agrobacterium-mediated transformation

## Abstract

*Eucalyptus* is a genus of over 900 species and hybrids, and many of them are valuable fast-growing hardwoods. Due to its economic importance, *Eucalyptus* is one of the early tree species whose genomes were deciphered. However, the lack of efficient genetic transformation systems severely restricts the functional genomic research on the plant. The success of *Eucalyptus* regeneration and transformation depends greatly on the genotypes and explants. In this study, we systematically screened 26 genotypes from 12 *Eucalyptus* species in an attempt to obtain *Eucalyptus* genotypes with high regeneration potential. We developed two common regeneration media that can be applied to most tested *Eucalyptus* genotypes for both seeding hypocotyls and cloned internodes as explants. We then implemented DsRed2 as a visual marker for genetic transformation efficiency test. Our results suggest that *E. camaldulen* and *E. robusta* are amenable for genetic transformation. Finally, we successfully set up a stable *Agrobacterium*-mediated genetic transformation procedure for both *E. camaldulen* and *E. robusta* using seeding hypocotyls and cloned internodes respectively. Taken together, our study provides valuable means for vegetative propagation, gene transformation, CRISPR based gene mutagenesis, activation and suppression, as well as functional characterization of genes in *Eucalyptus*.

## INTRODUCTION

Eucalypts (*Eucalyptus*, Myrtaceae) are the world's most valuable fast-growing, broad-leaved hardwood trees. They are native to Australia and have been widely introduced around the world due to their economic importance, such as serving as a source of timber, paper pulp, and essential oils^[1,2]^. The genus *Eucalyptus* comprises more than 900 species and hybrids, which provide a rich genetic diversity base for *Eucalyptus* genetic breeding^[2]^. However, the traditional genetic breeding of *Eucalyptus* is limited by a long period of breeding cycles, high levels of heterozygosity, and difficulty of hybridization^[3]^. The genetic modification (GM)-based transgene technology not only enables the introduction of specific traits of interest into a desirable genotype in a single generation, but also enables overcoming reproductive barriers by transferring selected genes across the genus^[4]^. Furthermore, with the release of the *Eucalyptus* *grandis* genome, transgenic technology has become increasingly important to study the basic questions of plant biology in woody perennials^[5]^. However, the lack of efficient genetic transformation seriously restricts functional genomic research on the plant^[3]^.

In the past two decades, various research groups have reported different genetic transformation protocols and attempted to develop transgenic eucalypts. Although genetic transformation protocols have been established for several *Eucalyptus* species^[6−11]^, the transformation system is unstable, and the efficiency is very low. The successful transformation of eucalypts depends on many factors, such as genotypes, explant types, *Agrobacterium* strains, culture media, and growth conditions^[12]^. However, most publications are confined only to one or two genotypes, and the protocols vary greatly, making the research hard to replicate^[12]^. This could explain why only few functional studies have been performed in transgenic eucalypts^[3]^. Recently, a hairy roots transformation system for *E. grandis* was set up^[13]^*.* Two wood-related genes were studied, which provides an optional tool to functionally characterize eucalypt genes^[14]^. However, this system is limited to organ-specific contexts and suitable for investigating root-specific processes and interactions^[14]^. Thus, we still need to make efforts to establish a stable and more efficient genetic transformation system of eucalypts.

The establishment of genetic transformation depends on an effective reporter system, which allows visual detection of transformed tissues. Numerous reporter genes including those encoding *β*-glucuronidase (GUS)^[15]^, fluorescent proteins (FPs)^[16,17]^, and luciferase (LUC)^[18]^ have been widely employed to visualize target gene expression or identify transgenic lines in diverse plant species. All of them have their own advantages and limitations. For instance, the *GUS* gene is one of the most widely used reporter genes in many plant species, including *Eucalyptus*^[19,20]^. It can be simply detected by histochemical staining but material is consumable due to the destructive nature of the staining and de-staining procedure. Moreover, high frequency results in false-positive happens in earlier callus selection due to its GUS activities in *Agrobacterium*^[21]^. Green fluorescent protein (GFP) from the *Auquorea victoria* jellyfish is another reporter widely used in several living organisms^[16]^. It can be measured simply using a fluorescence detector without additional substrates or cofactors^[17,22]^. However, it has high autofluorescence^[23]^, and cytotoxicity and immunogenicity due to GFP aggregates^[ 17, 24−26]^. The *LUC* genes mainly appliable to monitor real-time gene expression^[27]^. Its measurement relies on additional substrate and bioluminescence, and depends largely on the local cell environment. Thus, it is rarely applied in the genetic transformation reporter system^[18,26]^. DsRed2 (*Discosoma* red fluorescent protein 2) is a DsRed mutant form of the oral disk of coral (*Discosoma* sp.), and its spectral characteristics are significantly different from those of GFP, with a much higher extinction coefficient and yield of fluorescence quantum^[28−30]^. DsRed2 is mostly used in animal imaging. Recently, DsRed2 has been used in various plant transgenic studies, such as studies on cotton^[31]^, tobacco^[32]^, rice^[33]^, soybean^[34]^, and walnut^[35]^. Its transient expression and stable transformation had no negative effects on plant development and morphogenesis^[32,33]^. Thus, DsRed2 might be a better alternative reporter for genetic transformation studies.

In fact, the regenerative capacity and response to culture conditions are highly genotype-dependent^[36,37]^. Thus, screening of eucalypt genotypes favorable for *Agrobacterium* infection and tissue regeneration is the most important step to establishing an efficient genetic transformation system. In this study, the organogenesis efficiency of 12 eucalypt species (including 26 genotypes) was systematically tested with sown hypocotyls and cloned internodes as explants. These eucalypt species were successfully introduced to China and have been widely used for conventional *Eucalyptus* breeding^[38]^. Their transformation efficiency was tested by using DsRed2 as a reporter. Based on the regeneration and transformation efficiency studies, we focused on *E. camaldulen* and *E. robusta* genotypes, and finally developed an efficient and stable *Agrobacterium*-mediated genetic transformation method for both.

## RESULTS

### Screening the genotypes of multiple eucalypt species with high regeneration potential

**Figure 1:**
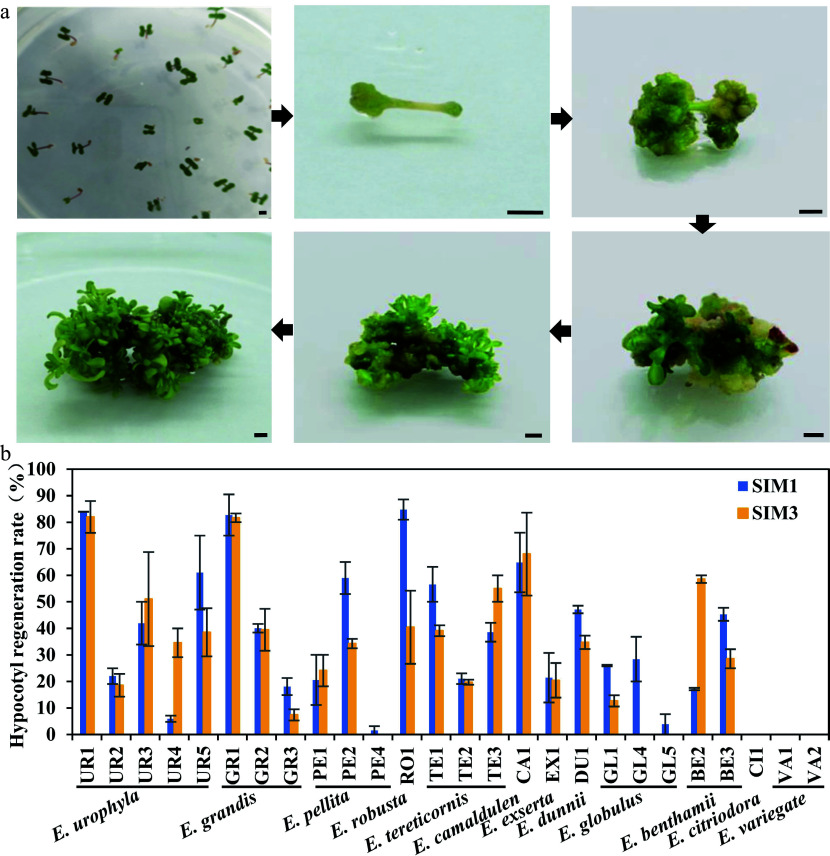
Regeneration efficiency of different eucalypt species and genotypes using seeding hypocotyl as explants. (a) Representative image of hypocotyl explants regeneration on SIM media at different stages. Photo were taken every other week. Bar = 2 mm. (b) Regeneration efficiency was recorded based on the regeneration ratio. The regeneration rate is defined as a/b × 100%, where a is the number of explants forming shoots after four weeks of screening, and b is the number of explants before the screening. Data shown are mean values from two biological replicates. Error bars ± SE.

Tissue regeneration capability is a crucial step in establishing an efficient genetic transformation system, while the regeneration capability is highly genotype-dependent. By integrating previous studies on eucalyptus regeneration media, four different shoot induction and multiplication media (SIM) were designed for initial regeneration screening in *E. camaldulen*, a eucalypt species with relatively high regeneration efficiency reported in previous studies^[3,12]^ (Supplemental Table S1). The results showed that *E. camaldulen* displayed high regeneration ability in two of the SIMs (SIM1 and SIM3). Thus, we used these two SIM media for further regeneration tests of other eucalypt species. Seeding hypocotyls of 12 eucalypt species (including 26 genotypes) were used as explants for the regeneration capability test. Following 4 weeks of culturing in SIM media, multiple shoots with tiny leaflets started to proliferate and enlarge from the nodal segment (Fig. 1a). The regeneration efficiency was then calculated. Most tested genotypes could be successfully regenerated in the two SIM media, except *E. variegate* and *E. citriodora* (Fig. 1b, Table 1). However, the regeneration efficiency varied greatly among species and even among different genotypes of the same species (Fig. 1b, Table 1). The regeneration efficiencies of many genotypes, including *E. urophyla* (UR1), *E. robusta* (RO1), *E. grandis* (GR1), and *E. camaldulen* (CA1), were up to 80% (Fig. 1b, Table 1), which were considered as good candidates for genetic transformation system establishment. Although the regeneration efficiency in SIM1 was slightly higher than that in SIM3, both SIM media were considered suitable regeneration media and could be applicable to multiple eucalypt species.

**Table 1:** Regeneration rate of shoots induced by hypocotyl as explants in different eucalyptus species or genotypes.

Species	Clone	SIM1					SIM3			
		Total	Shoot induction	Regeneration efficiency (%)	Differentiation amount		Total	Shoot induction	Regeneration efficiency (%)	Differentiation amount
*E.urophylla*	UR1	81	68	83.96 ± 0.04a	***		50	41	82.00 ± 6.00^a^	***
	UR2	41	9	22.02 ± 2.98^ghi^	*		56	11	18.57 ± 4.29^ijkl^	*
	UR3	78	29	41.94 ± 8.06^def^	***		67	28	51.04 ± 17.71^bcde^	***
	UR4	35	2	5.95 ± 1.19^ijk^	*		44	15	34.58 ± 5.42^efghi^	**
	UR5	33	20	61.03 ± 13.97^bc^	*		38	15	38.52 ± 9.10^defg^	*
*E.grandis*	GR1	53	43	82.74 ± 7.74^a^	***		49	40	81.67 ± 1.67^a^	**
	GR2	87	35	40.06 ± 1.60^def^	**		38	47	39.47 ± 7.89^cdef^	*
	GR3	162	28	18.08 ± 3.23^hij^	*		80	6	7.39 ± 2.13^kl^	*
*E.pellita*	PE1	28	5	20.56 ± 9.44^hi^	**		21	5	24.09 ± 5.91^fghijk^	**
	PE2	71	40	58.97 ± 6.03^bc^	**		65	22	34.25 ± 1.75^efghi^	**
	PE4	43	1	1.56 ± 1.56^jk^	*		66	0	0.00 ± 0.00^l^	
*E.robusta*	RO1	112	96	84.76 ± 3.81^a^	***		87	43	40.42 ± 13.75^cdef^	***
*E.tereticornis*	TE1	47	26	56.58 ± 6.58^bcd^	***		44	17	39.11 ± 2.07^defg^	**
	TE2	60	13	21.06 ± 2.01^hi^	**		61	12	19.72 ± 0.97^hijk^	*
	TE3	39	15	38.55 ± 3.55^efg^	**		40	22	55.00 ± 5.00^bcd^	***
*E.camaldulen*	CA1	102	65	64.83 ± 11.26^b^	***		76	57	68.01 ± 15.63^ab^	***
*E.exserta*	EX1	85	20	21.45 ± 9.32^hi^	*		62	12	20.41 ± 6.52^ghijk^	**
*E.dunnii*	DU1	186	88	47.13 ± 1.45^cde^	**		192	67	34.74 ± 2.52^efghi^	**
*E.globulus*	GL1	54	14	26.02 ± 0.30^fgh^	*		65	8	12.67 ± 2.14^jkl^	*
	GL4	107	31	28.42 ± 8.42^fgh^	*		99	0	0.00 ± 0.00^l^	
	GL5	98	5	3.85 ± 3.85^jk^	*		92	0	0.00 ± 0.00^l^	
*E.benthamii*	BE2	190	33	17.12 ± 0.45^hij^	**		27	16	58.57 ± 1.43^bc^	**
	BE3	79	36	45.29 ± 2.44^cde^	***		88	26	28.57 ± 3.57^fghij^	**
*E.citriodora*	CI1	122	0	0.00 ± 0.00^k^			92	0	0.00 ± 0.00^l^	
*E.variegate*	VA1	123	0	0.00 ± 0.00^k^			109	0	0.00 ± 0.00^l^	
	VA2	125	0	0.00 ± 0.00^k^			116	0	0.00 ± 0.00^l^	
Mean values of two independent experiments ( ± ) with standard errors. Values with the same letter within columns are not significantly different according to Duncan’s Multiple Range Test (DMRT) at a 5% level. Shoots number of each explant were counted and classified (*, < five shoots; five shoots < ** < ten shoots; ***, > ten shoots).										

### Regeneration capacity test of internode explants

**Figure 2:**
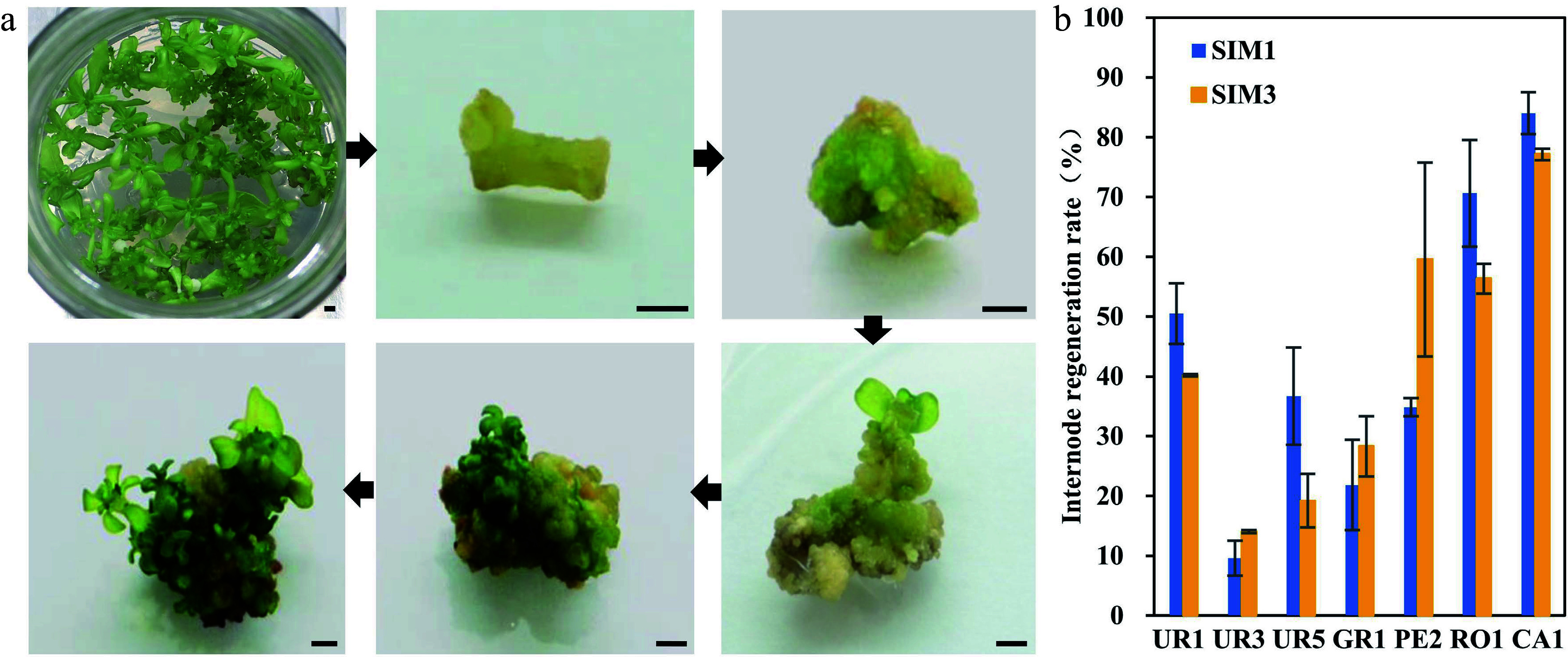
Regeneration efficiency of different eucalyptus species and genotypes using clonal internode as explants. (a) Representative image of clonal internode explants regeneration on SIM media at different stages. Photos were taken every other week. Bar = 2 mm. (b) Regeneration efficiency was recorded based on the regeneration ratio. The regeneration rate is defined as a/b × 100%, where a is the number of explants forming shoots after four weeks of screening, and b is the number of explants before the screening. Data shown are mean values from two biological replicates. Error bars ± SE.

Seeding tissues, such as hypocotyl or cotyledon, are usually good explants for *in vitro* regeneration. However, since* Eucalyptus* is an outcrossing tree species, the offspring have great genetic variation, which will make transformation unstable and count against future transgenic analysis. Thus, clones from the seven highest regeneration efficiency eucalypt species or genotypes were chosen and sub-cultured. The internodes of these clones were used as explants for the regeneration efficiency test (Fig. 2a). The results showed that all test clones could be successfully regenerated (Fig. 2b, Table 2). In general, we found that the regeneration efficiency of internode explants was lower compared to that of hypocotyl explants (Fig. 2b, Table 2). Among them, *E. urophyla* (UR1), *E. pellita* (PE2), *E. robusta* (RO1), and *E. camaldulen* (CA1) displayed better regeneration ability, with regeneration efficiency over 50%.

**Table 2:** Regeneration rate of shoots induced by stem segments as explants in different eucalypt species or genotypes.

Species	Clone	SIM1					SIM3			
		Total	Shoot induction	Regeneration efficiency (%)	Differentiation amount		Total	Shoot induction	Regeneration efficiency (%)	Differentiation amount
*E.urophylla*	UR1	80	40	50.51 ± 5.05^b^	***		92	37	40.19 ± 0.19^bc^	**
	UR3	46	4	9.58 ± 2.92^d^	**		36	5	14.04 ± 0.25^d^	*
	UR5	57	21	36.70 ± 8.13^bc^	*		71	14	19.20 ± 4.49^cd^	*
*E.grandis*	GR1	41	11	21.85 ± 7.56^cd^	**		49	12	28.29 ± 5.04^cd^	***
*E.pellita*	PE2	70	24	34.85 ± 1.52^bc^	**		63	38	59.55 ± 16.21^ab^	**
*E.robusta*	RO1	125	85	70.63 ± 8.91^a^	**		111	64	56.33 ± 2.49^ab^	***
*E.camaldulen*	CA1	117	97	84.01 ± 3.49^a^	***		74	57	77.16 ± 0.97^a^	**
Mean values of two independent experiments ( ± ) with standard errors. Values with the same letter within columns are not significantly different according to Duncan's Multiple Range Test (DMRT) at a 5% level. Shoots number of each explant were counted and classified (*, < five shoots; five shoots < ** < ten shoots; ; ***, > ten shoots).										

### Transformation efficiency determination

**Figure 3:**
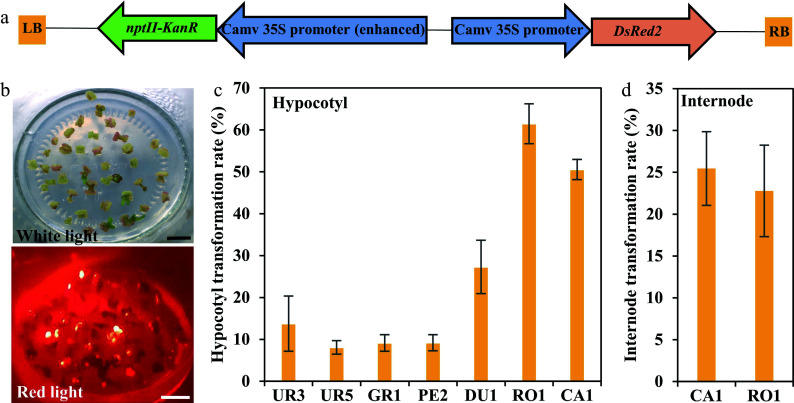
Transformation efficiency of different eucalyptus species and genotypes. (a) T-DNA region of pCAMBIA2300::35S::DsRed2 vector for genetic transformation. The chimeric neomycin phosphotransferase II (NPT II) selection marker and the reporter gene DsRed2 were driven by cauliflower mosaic virus 35S promoter. LB and RB indicate T-DNA left and right border, respectively. Arrows indicate the direction of transcription. (b) Fluorescence observation of callus. Callus induction one month after Agrobacterium infection were observed at white light and red light with the fluorescence stereo-microscope. Bar = 1 cm. (c), (d) Transformation efficiency calculation of different eucalyptus species and genotypes using seeding hypocotyl and clonal internode as explants, respectively. Transformation efficiency was recorded based on the fluorescence callus of each explant. The transformation rate is defined as a/b × 100%, where a is the number of explants having fluorescence callus after one month of screening, and b is the total number of explants. Data shown are mean values from two biological replicates. Error bars ± SE.

Based on previous studies, transformation efficiency is largely dependent on genotypes. To access the *Agrobacterium* susceptibility of different eucalypt genotypes, *A. tumefaciens* strain GV3101 (pMP90) that harbored the binary vector pCAMBIA2300 containing the *DsRed2* reporter gene under the control of the 35S was used for transformation (35S::*DsRed2*, Fig. 3a). DsRed2 encodes a red fluorescent protein that can be detected under fluorescence microscopy and has been widely used in genetic transformation^[31−35]^. We first performed the *Agrobacterium*-mediated genetic transformation test of the seven highest regeneration efficiency eucalypt species with hypocotyls as explants. The transformation efficiency was calculated based on DsRed2 fluorescence on the callus (Fig. 3b). The results showed that the transformation efficiency varied greatly among different eucalypt species (Fig. 3c, Table 3). *E*. *urophylla* (UR1 and UR5), *E. grandis* (GR1), and *E. pellita* (PE2) displayed quite low transformation efficiency, with few visible red fluorescent calli. By contrast, more than half of the RO1 and CA1 explants had fluorescent calli (Fig. 3c, Table 3), suggesting a higher susceptibility to *Agrobacterium*. We further used internodes from established clones of RO1 and CA1 as transformation explants. However, the transformation efficiency of clonal internodes decreased to 20%, with half the efficiency compared to that using seeding hypocotyls as explants (Fig. 3d, Table 3). These results suggest that the transformation efficiency is highly dependent on the explant types.

**Table 3:** Transformation efficiency test of different eucalypt species by monitoring the red fluorescence rate of the callus after transformed with the reporter DsRed2.

Species	Clone	Explant	Total	Red fluorescence	Transformation efficiency (%)
*E. robusta*	RO1	Hypocotyl	177	89	61.48 + 4.76^a^
*E. camaldulen*	CA1	Hypocotyl	71	31	50.55 + 2.40^a^
	CA2	Hypocotyl	63	19	29.68 + 2.76^b^
*E. dunnii*	DU1	Hypocotyl	159	45	27.30 + 6.40
*E. urophylla*	UR3	Hypocotyl	73	13	13.75 + 6.60^c^
	UR4	Hypocotyl	93	8	8.07 + 1.62^c^
*E. pellita*	PE2	Hypocotyl	73	6	9.19 + 1.92^c^
*E. grandis*	GR1	Hypocotyl	41	4	9.13 + 1.98^c^
*E. robusta*	RO1	Internode	48	11	22.78 + 5.47^a^
*E. camaldulen*	CA1	Internode	475	124	25.46 + 4.40^a^
Mean values of two independent experiments (±) with standard errors. Values with the same letter within columns are not significantly different according to Duncan’s Multiple Range Test (DMRT) at a 5% level.					

### Stable transformation system establishment of *E. camaldulen* and *E. robusta*

**Figure 4:**
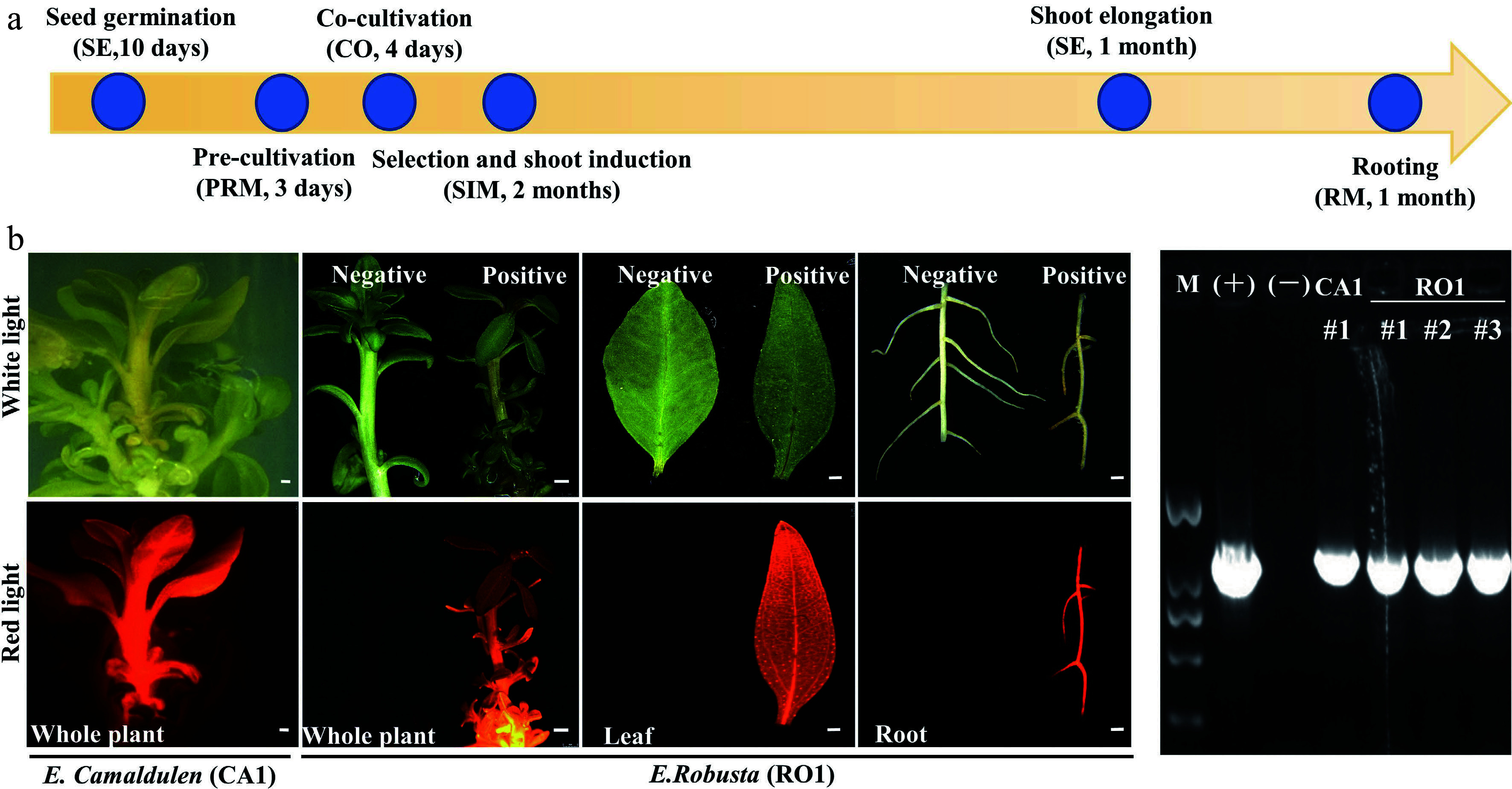
Agrobacterium-mediated eucalyptus genic transformation with DsRed2 as reporter gene. (a) Flow diagram for agrobacterium-mediated eucalyptus genic transformation. The yellow arrow represents the process direction of transformation. The blue circles with notes represent important transformation steps and time required (the media information of each step is listed in Supplemental Table S2). (b) DsRed2 gene expression in different tissue and organs in transgenic plants. Photos were taken at white light and red light with the fluorescence stereo-microscope. *E. robusta* (RO1) plants that untransformed were used as a negative control (Negative). Successful transformed plants were marked with Positive. Bar = 1 mm. (c) Positive test of DsRed2 transgenic plants using PCR amplification. M, DNA marker; (+), Positive control with transformation vector as the PCR template; (−), wild type plants; CA1 and RO1, genotypes of *E. camaldulen* and *E. robusta* respectively used for transformation donor in this study*.* The number represents the independent transgenic line.

Combined with the results of the regeneration and transformation tests, we selected CA1 and RO1 for stable transformation system establishment. Seeding hypocotyls of CA1 and RO1 were used as explants and were transformed by *A. tumefaciens* strain GV3101 (pMP90) harboring the DsRed2 overexpression construct. It has been shown that pre-culture and co-culture before and after *Agrobacterium* infection will improve transformation efficiency^[12]^. In our early trials, the combination of 3-day pre-culture prior to inoculation and 4-day co-cultivation after inoculation was ideal for *Agrobacterium* infection (Fig. 4a). After 8 weeks on selection and shoot regeneration medium (SIM1) and a further 4 weeks on shoot elongation medium (SE), adventitious shoots formed. The adventitious shoots were then transferred to the rooting media (RM) to obtain complete transgenic plants (Fig. 4a). The positive plants were screened based on DsRed2 fluorescence and PCR tests (Fig. 4b, c). We were able to obtain positive transgenic plants in all three test batches (Fig. 4b, Table 4). Furthermore, we applied this transformation system by overexpressing the eucalypt flowering time gene* EgFT* using clonal internode segments as explants. We obtained positive plants in every single transformation batch (Table 4). This result indicated that we successfully established a stable transformation system of both *E. camaldulen* and *E. robusta* using either hypocotyls or stem segments as explants*.* Although the transformation efficiency was still low, such a stable transformation system will help us optimize the transformation procedure in the future.

**Table 4:** Transformation efficiency test of *E.robusta* and* E.camaldulen* using hypocotyl and internode as explants respectively.

Genotype	Construct	Explant type	Explant number	Positive plants	Transformation efficiency (%)
*E. robusta* (RO1)	35S::*DsRed*	Hypocotyl	77	3	3.9
*E. robusta* (RO1)	35S::*DsRed*	Hypocotyl	67	1	1.5
*E. camaldulen* (CA1)	35S::*DsRed*	Hypocotyl	55	1	1.9
*E. robusta* (RO1)	35S::*EgFT*	Internode	51	2	3.9
*E. camaldulen* (CA1)	35S::*EgFT*	Internode	69	1	1.5

## DISCUSSION

Stable transformation protocols were successfully established for several *Eucalyptus* species by various research groups around the world^[3,19,39,40]^. However, the genetic transformation of *Eucalyptus* is still in its infancy. Except for a few commercial genotypes, there still lack widely applicable protocols for eucalypt transformation as it is too time-consuming and has low efficiency. For these reasons, very few functional studies have been performed in transgenic *Eucalyptus* (reviewed in Girijishankar^[3]^). Successful plant stable genetic transformation depends on various factors, such as genotype, type of explant, medium composition, culture conditions, and so on. Among them, genotype is the most influential factor in *Eucalyptus* genetic transformation. To our knowledge, there are rare reports focused on genotype selection in eucalypt transformation tests. In this study, we screened as many as 26 eucalypt species or genotypes to find eucalypt species or genotypes harboring both high efficiency of regeneration and transformation. Based on this large-scale screening, we developed common regeneration media that were suitable for testing on most eucalypt species, either using hypocotyls or clonal internodes as explants. We also obtained eucalypt genotypes that were highly susceptible to *Agrobacterium*, which improves transformation efficiency. Our results showed that *E. camaldulen* and *E. robusta* display the best regeneration and transformation performance among all testing eucalypt species. In addition, although the regeneration or transformation efficiency of *E*. *dunnii* (DU1) is not the best, it has great potential to be a genetic transformation receptor (Fig. 1b & 3c). In this study, besides* E. camaldulen,* which has been studied previously, we first reported that *E. robusta* had great potential for further *Eucalyptus* transformation study. Furthermore, we successfully set up a stable genetic transformation procedure for both *E. camaldulen* and *E. robusta* using seeding hypocotyls or cloned internodes. The procedure is stable and efficient, with the highest transformation frequency of up to 3.9%. Overall, our study provides a valuable tool for the study of *Eucalyptus* functional genomics and molecular breeding.

Besides genotypes and explants, we noticed that a resistance selection agent is crucial for shoot regeneration after transformation. We used neomycin phosphotransferase (NPT II) selection systems in our study. In previous studies, the screening concentration of kanamycin varied from 10 mg L^−1^ to 90 mg L^−1^ due to different antibiotic tolerance among *Eucalyptus* species^[6,11]^. In this study, shoots were almost completely prevented when treated with 40 mg L^−1^ kanamycin, while many shoots were regenerated from explants treated with 30 mg L^−1^ kanamycin. Thus, we set 30 mg L^−1^ kanamycin as the screening pressure in our study. However, although *E. camaldulen* and *E. robusta* have as high as 80% shoot regeneration efficiency and up to 50% transformation rates, the regeneration rate dramatically decreased after agrobacterial transformation and kanamycin selection. Only few explants regenerated putatively transformed shoots. In the future, optimization of selection conditions could prove to be an effective method of improving *E. camaldulen* and *E. robusta* transformation efficiency.

An appropriate reporter gene can effectively help in the detection of transformed cells. In *Eucalyptus*, the *GUS* gene has been used as a reporter gene. However, it depends on histochemical staining, which demands tissue destruction. Moreover, it was reported that transgenic callus with GUS expression could not regenerate into shoots in *Eucalyptus*^[40]^. In this study, we applied DsRed2 as a reporter in eucalyptus transformation efficiency screening and stable transformation system setup. It can visually distinguish transgenic from non-transgenic callus of *Eucalyptus* at an early stage (Fig. 3b) and is expressed in almost all plant tissues without organ preference (Fig. 4b). The application of DsRed2 in this study suggests that DsRed2 is an ideal morphological reporter for *Eucalyptus* genetic transformation establishment and further transformation efficiency improvement.

## CONCLUSIONS

In this study, we systematically screened the regeneration and *Agrobacterium*-mediated transformation competence of eucalypt species or genotypes on a large scale. We developed common regeneration media that are suitable for testing on most eucalypt species. We also obtained high *Agrobacterium* susceptibility amenable eucalypt genotypes, which will help to improve transformation efficiency. Finally, we set up a stable and efficient *Agrobacterium*-mediated genetic transformation procedure for both *E. camaldulen* and *E. robusta* using both hypocotyls and clonal internodes as explants. Overall, our study provides powerful means for eucalyptus propagation, genomics research and molecular breeding.

## MATERIALS AND METHODS

### Plant material, strains, and plasmids

Seeds of *Eucalyptus* in this study were provided by the China Eucalypt Research Centre (Zhanjiang, China). *A. tumefaciens* GV3101 and competent cells of *E. coil* DH5α were homemade in this laboratory. The pCAMBIA2300::35S::DsRed2 vector plasmids were kindly provided by Professor Shuangxia Jin, Huazhong Agricultural University^[31]^. For the 35S::*EgFT* construct, full-length cDNA of *EgFT* (*Eucgr.B01458*) was amplified from *E. grandis*. The fragments were then transformed to the destination vector pK2GW7^[41]^.

### Media and culture conditions

The Murashige and Skoog (MS) basal medium with 20 g L^−1^ sucrose and 0.8% (w/v) bacteriological agar was used in all media in this study^[42]^. The pH of the medium was adjusted to 5.8 and autoclaved at 121 °C for 20 min. Filter-sterilized phytohormones were added to the medium after autoclaving. For shoot regeneration, cultures were maintained under a photoperiod of 16 h light and 8 h dark with a light intensity of 12 μmol m^−2^ s^ −1^ provided using white fluorescent tube lights. For shoot elongation and rooting, the light intensity was increased to 55 μmol m^−2^ s^ −1^. The room temperature was maintained at 24 ± 2 °C during the whole culture process.

### Explant preparation

For hypocotyl explants, seeds were washed three times in distilled water, surface-sterilized in 70% (v/v) ethanol for 1 min and 1.5% (v/v) NaClO for 15 min with constant stirring, followed by four washes in sterile distilled water. Washed seeds were protected from light in Germination Medium (GM) at 25 °C for 3 d and then transferred to a 16 h light and 8 h dark growth chamber. Hypocotyls were cut for the regeneration experiment. For internode explants, shoot clones regenerated from hypocotyls of different species or genotypes were sub-cultured in SIM. Internodes from the shoots were then cut for the regeneration and transformation experiments.

### *Agrobacterium*-mediated transformation

*A. tumefaciens* strain GV3101 harboring the pCAMBIA2300::35S::DsRed2 binary vector was used for the transformation experiments. The strains were inoculated in liquid YEB (Yeast Mannitol Medium) medium containing 25 mg L^−1^ rifampicin and 100 mg L^−1^ spectinomycin and grown at a temperature of 28 °C on a shaker at 200 rpm until OD_600_ = 0.5–1.0. The bacteria were resuspended in liquid pre-cultivation medium (PRM). The explants of hypocotyls or internodes were cut into 5-mm slices and dipped in the bacterial suspension for 30 min. The explants were dried on filter paper and subsequently transferred onto a co-cultivation medium. The plates were incubated for 3 days in dark conditions at 24 °C. For selection, the explants were transferred to a solidified selection and SIM. Two months later, the regenerated shoots were transferred into a shoot elongation (SE) medium until they were suitable for rooting. Elongated shoots were excised and placed in a rooting medium (RM). After 2 weeks, the roots were fully developed, and the plants could eventually be transplanted in the pots grown in the greenhouse. The media used in this study are listed in Supplemental Table S2.

### Regeneration and transformation efficiency determination

For the regeneration efficiency test, the explants were incubated in different SIMs (SIM1 and SIM3, Supplemental Table S1). After incubation for 28 d in the culture room, the number of shoots per explant, and the percentage (%) of explants forming shoots were measured and recorded. The regeneration rate is defined as a/b × 100%, where a is the number of explants forming shoots after 4 weeks of screening, and b is the number of explants before the screening. The regeneration capability was also monitored by counting the number of shoots of each explant and classified into three types (i.e., high, > ten shoots; ten shoots > medium > five shoots; and low, < five shoots).

In this study, we used the red fluorescent protein DsRed2 as a reporter to check eucalyptus transformation efficiency. Calli from the regenerated explants were observed under white light and red light with a fluorescence stereomicroscope (Olympus, Tokyo, Japan). The red light uses a filter set for excitation at 530−550 nm and emission at 575 nm. The transformation rate is defined as a/b × 100%, where a is the number of explants that contain red florescence light after four weeks of screening, and b is the number of explants before the screening.

### Molecular analysis

Genomic DNA was extracted from young leaves of putative transgenic shoots and wild eucalypt shoots using a Plant Genomic DNA Kit (Tiangen Biotech, Beijing, China). Gene-specific primers of* DsRed2* and *EgFT* were designed and used for positive checking. The construction vectors used for transformation served as a positive control, while DNA from untransformed plants served as a negative control. The amplification products were separated by electrophoresis on a 1.0% (w/v) agarose gel and visualized using a UV transilluminator. Primers used in this study are listed in Supplemental Table S3.

## SUPPLEMENTARY DATA

Supplementary data to this article can be found online.
