# Supplementary material for: Regeneration and Agrobacterium-mediated genetic transformation of twelve Eucalyptus species
Source: For Res (Fayettev). 2022 Nov 24;2:15. doi: 10.48130/FR-2022-0015 (PMC11524307; doi:10.48130/FR-2022-0015)
Supplement: Supplementary file 1 — Supplementary data to this article can be found online. [file FR-2022-0015-S1.zip › 10.48130_FR-2022-0015-Suppl-TableS2.pdf]

Supplementary Table S2. The media used in this study.

Medium	Composition
YEB medium	10 g/L tryptone, 10 g/L NaCl, 5 g/L yeast extract, pH7.0
Seed germination (GM)	1/2MS, 7 g/L agar, pH5.8
Pre-cultivation (PRE)	MS, 20 mg/L sucrose, 0.2 mg/L BAP, 0.05 mg/L NAA, 14% MES, 10μM acetosyringone, 6g/L agar, pH5.6
Co-cultivation (CO)	MS, 20 mg/L sucrose, 0.2 mg/L BAP, 0.05 mg/L NAA, 14% MES, 6g/L agar, pH5.6
Selection (SIM)	MS, 20 mg/L sucrose, 0.2 mg/L BAP, 0.05 mg/L NAA, 14% MES, 30 mg/L Kanamycin, 300 mg/L cefotaxime, 6 g/L agar, pH5.6
Shoot Elongation (SE)	MS, 20 mg/L sucrose, 0.1 mg/L BAP, 0.1 mg/L NAA, 14% MES, 6 g/L agar, pH5.8
Rooting (RM)	MS, 20 mg/L sucrose, 0.2 mg/L IBA, 6 g/L agar, pH5.8
