# Supplementary material for: Regeneration and Agrobacterium-mediated genetic transformation of twelve Eucalyptus species
Source: For Res (Fayettev). 2022 Nov 24;2:15. doi: 10.48130/FR-2022-0015 (PMC11524307; doi:10.48130/FR-2022-0015)
Supplement: Supplementary file 1 — Supplementary data to this article can be found online. [file FR-2022-0015-S1.zip › 10.48130_FR-2022-0015-Suppl-TableS3.pdf]

Supplementary Table S3. Primers used in this study.

Primer name	Sequences
DsRed-Forward	CCATGGAGTCAAAGATTCAA
DsRed-Reverse	GGCCAATCCAGAAGATG
EgFT-Forward	ACGCACAATCCCACTATCCTTC
EgFT-Reverse	GCGGACTCTAGCATGGCCGC
