# Supplementary material for: Regeneration and Agrobacterium-mediated genetic transformation of twelve Eucalyptus species
Source: For Res (Fayettev). 2022 Nov 24;2:15. doi: 10.48130/FR-2022-0015 (PMC11524307; doi:10.48130/FR-2022-0015)
Supplement: Supplementary file 1 — Supplementary data to this article can be found online. [file FR-2022-0015-S1.zip › 10.48130_FR-2022-0015-Suppl-TableS1.pdf]

Supplementary information:

Supplementary Table S1. Shoot induction and multiplication media (SIM) used for regeneration test.

Treatments	Basic culture medium	Plant growth regulator ($mg·L^{-1}$)				Regeneration capability
		**BAP**	**IBA**	**TDZ**	**NAA**	
SIM1	MS	0.2			0.05	***
SIM2	MS	0.2			2	**
SIM3	MS	0.2	0.1	0.03		***
SIM4	MS	0.2	0.1	0.01		**

BAP, 6-Benzyle aminopurine; IBA, Indole-3-butyric acid; TDZ, Thidiazuron; NAA, 1-naphthaleneacetic acid. Shoots number of each explant were counted (***, >ten shoots; **, <ten shoots).
